# IL-15 Participates in the Respiratory Innate Immune Response to Influenza Virus Infection

**DOI:** 10.1371/journal.pone.0037539

**Published:** 2012-05-18

**Authors:** Katherine C. Verbist, David L. Rose, Charles J. Cole, Mary B. Field, Kimberly D. Klonowski

**Affiliations:** 1 Department of Cellular Biology, University of Georgia, Athens, Georgia, United States of America; 2 Department of Infectious Diseases, University of Georgia, Athens, Georgia, United States of America; MRC National Institute for Medical Research, United Kingdom

## Abstract

Following influenza infection, natural killer (NK) cells function as interim effectors by suppressing viral replication until CD8 T cells are activated, proliferate, and are mobilized within the respiratory tract. Thus, NK cells are an important first line of defense against influenza virus. Here, in a murine model of influenza, we show that virally-induced IL-15 facilitates the trafficking of NK cells into the lung airways. Blocking IL-15 delays NK cell entry to the site of infection and results in a disregulated control of early viral replication. By the same principle, viral control by NK cells can be therapeutically enhanced via intranasal administration of exogenous IL-15 in the early days post influenza infection. In addition to controlling early viral replication, this IL-15-induced mobilization of NK cells to the lung airways has important downstream consequences on adaptive responses. Primarily, depletion of responding NK1.1+ NK cells is associated with reduced immigration of influenza-specific CD8 T cells to the site of infection. Together this work suggests that local deposits of IL-15 in the lung airways regulate the coordinated innate and adaptive immune responses to influenza infection and may represent an important point of immune intervention.

## Introduction

Influenza virus is a major human pathogen that causes substantial morbidity and mortality—approximately 36,000 deaths annually in the United States alone [1]. Combined with the severe economic burden imposed from seasonal influenza outbreaks and growing concerns over potential imminent influenza pandemics, there is considerable need for a firm understanding of the disease pathology, prevention strategies, and mechanisms of host defense against the virus [2].

Influenza virus is primarily transmitted via inhaled aerosols and results in an infection localized to the upper respiratory tract, with viral replication largely limited to epithelial cells [3]. Mechanisms by which the immune system eliminates influenza have been well studied and are known to involve the coordinated actions of the innate and adaptive immune systems. Namely, the cytolytic action of influenza-specific CD8 T cells has been shown to be the primary mediator of complete viral clearance, but important roles have also been described for CD4 T cells [4], [5], [6]. In addition to T cells, a crucial role has also been established for innate immune effectors including natural killer (NK) cells, which provide short-term control of viral replication prior to T cell activation [7]. NK cells become activated following the loss of inhibitory signals coupled with positive activating signals resulting in direct (via release of cytotoxic granules and interferon γ) or indirect (via activation of macrophages and dendritic cells) target cell lysis [8]. NK cells are vital in limiting influenza viral replication as depletion of NK cells dramatically increases morbidity and mortality in hamsters and mice [9], and in humans severe infections with the 2009 pandemic H1N1 virus positively correlated with reduced numbers of NK cells in the lungs [10]. Studies have indicated that the natural cytotoxicity receptors NKp44 and NKp46, which recognize hemagglutinin proteins of several different influenza strains [11], [12] is one mechanism used by NK cells to protect against lethal viral challenge [13]. Secondarily, NK cells also aid in viral clearance indirectly through the production and secretion of cytokines which both amplifies local inflammation and recruits antigen-specific CD8 T cells to sites of inflammation [14]. Implicit in both of these functions is the ability of NK cells to accumulate within the respiratory tract to contact infected cells and provide a source of chemotactic signals to recruit recently activated CD8 T cells.

Type I IFNs expressed within hours after viral infection have been documented to induce expression of the chemokines CXCL9 and 10 which function to recruit CXCR3 expressing NK cells to sites of infection [15]. However, Type I IFNs also modulate the expression of the common gamma chain cytokine interleukin 15 (IL-15) [16], [17], [18], which we recently reported to be temporally and locally increased following influenza infection [19]. This expression of IL-15 in the respiratory tract facilitates the recruitment of antigen-specific CD8 T cells to the respiratory tract. However, it is unclear whether the chemotactic properties of IL-15 uniquely affect migratory CD8 T cells or could be extended to other IL-15-sensitive immune cells. NK and NKT cells are nearly absent in IL-15^−/−^ animals [20], highlighting the important role of IL-15 on NK cell development and homeostasis in the steady-state. Following viral infections, de novo production of IL-15 by dendritic cells results in the activation and proliferation of NK cells [21], [22], and transient systemic stimulation of NK cells with soluble IL-15/IL-15Rα complexes also results in an accumulation of phenotypically and functionally mature NK cells [23], [24]. In addition to these roles, IL-15 also can stimulate the migration of NK cells *in vitro* and enhances their adhesion to cultured endothelial cells [25]. We therefore hypothesized that virally induced IL-15 functionally assists in the migration of NK cells into the lung airways. We show here that an IL-15 deficiency results in a site-specific reduction in NK cells from the lung airway and an exacerbation of viral load at early time points post influenza infection. Additionally, exogenous IL-15 induces the specific migration of NK cells in vitro and in vivo. This IL-15-dependent enhanced mobilization of NK cells to the lung airways correlates with decreased viral loads. Importantly, in the absence of NK cells, antigen-specific CD8 T cells fail to accumulate at the site of infection, providing a possible link between IL-15-mediated migratory effects of both the innate and adaptive immune responses to influenza infection and suggest therapeutic possibilities regarding the use of IL-15 to simultaneously regulate both arms of the immune system for improved responses to viral infection.

## Materials and Methods

### Ethics Statement

All animals were handled in strict accordance with good animal practice as defined by the American Association for Accreditation of Laboratory Animal Care as well as federal and state agencies. All animal work presented here was approved by Institutional Animal Care and Use Committee of University of Georgia (AUP No. A2009-6-114).

### Mice, Viruses, IL-15 Blocking, and NK Cell Depletion

C57BL/6 mice were purchased from Charles River (Wilmington, MA) through the NCI program. Influenza A/HK-x31 (x31, H3N2) was generously donated by Dr. S. Mark Tompkins (University of Georgia, Athens, GA). Animals were infected intranasally (i.n.) with 10^3^ PFU HKx31 diluted in 50 µL sterile PBS. IL-15 was blocked using 25 µg anti-IL-15 mAb (clone AIO3) (eBioscience, San Diego, CA) administered daily via intraperitoneal injection (i.p.) in 200 µL sterile PBS. IL-15 depletion was confirmed by reductions in frequencies of both NK and CD44^hi^ CD8^+^ T cell in antibody-treated animals compared to untreated animals 7 days after the initiation of mAb treatment. NK1.1+ cells were depleted via intravenous (i.v.) injections of 200 uL PBS containing 200 µg anti-NK1.1/mouse (clone PK136) every other day (UCSF monoclonal antibody core facility, San Francisco, CA), and depletion of NK cells was verified using an anti-NKp46 mAb (Clone 21A9.4, eBioscience) throughout the experiment and 2 days after the last injection of the NK1.1 depleting mAb.

### Tissue Preparation and Flow Cytometry

Lung airway-resident cells were harvested by bronchioalveolar lavage (BAL) with 3 consecutive washes of 1 mL PBS. To isolate cells from the lung parenchyma, lungs were perfused with ∼25 mL PBS/heparin sodium solution, harvested, minced and incubated at 37°C for 30 minutes in 1.25 mM EDTA. The tissue was subsequently incubated in collagenase diluted in RPMI (6 mg/mL) at 37°C for 1 hour and passed through a 5 µm cell strainer. Isolated cells were subjected to separation via density gradient centrifugation by resuspending cells in 47% Percoll underlain with 67% Percoll. The gradients were then centrifuged at 2800 rpm for 20 minutes, and lymphocytes at the interface were collected. Spleen and lymph nodes were harvested from animals, homogenized, and then passed through a cell strainer. Spleen homogenate was depleted of erythrocytes by incubation in tris-buffered ammonium chloride.

Single cell suspensions were stained with combinations of cocktails containing anti-CD3, NK1.1, NKp46, CD11c, CD11b, CD122, and CD132 (eBioscience, San Diego, CA) as indicated for 20 minutes at 4°C. Where indicated, cells were concurrently stained with or without biotinylated anti-IL-15Rα (R&D Systems, Minneapolis, MN) followed by 20 minute incubation with APC-conjugated Streptavidin at 4°C. Influenza nuclear protein (NP) MHC class I tetramer [H2-D^b^/ASNENMETM] were generated by the National Institute of Allergy and Infectious Diseases Tetramer Facility (Emory University, Atlanta, GA). Tetramer staining was conducted at room temperature for 1 hour concurrently with anti-CD3, NK1.1, NKp46, CD8, and CD44 (eBioscience, San Diego, CA). Stained cells were analyzed using a BD LSRII digital flow cytometer (BD Biosciences, San Jose, CA) and either BD FACSDiva or FlowJo software (Tree Star, Inc., Ashland, OR).

### Migration and Proliferation Assays

IL-15 complexes (IL-15c) were generated on the day of use by incubating 1.5 µg recombinant mIL-15 with 7 µg IL-15Rα Fc-chimera (R&D Systems, Minneapolis, MN) at 37°C for 20 minutes followed by 4°C for at least 10 minutes. For Rα only controls, 7 µg IL-15Rα Fc-chimera (R&D Systems, Minneapolis, MN) was incubated similarly to complexes without addition of the cytokine. Complexes or Rα alone were administered via passive inhalation into both nostrils using a micropipette delivering 36.25 µL (for daily treatments) or 72.5 µL (for one time treatments) of complexes in sterile PBS. For assessment of cell proliferation, animals received 2 mg of BrdU (Sigma-Aldrich, St. Louis, MO) administered i.p. in a 200 µL volume of PBS. Cells were isolated from these animals 12 hours after treatment and stained with 20 µL aminoactinomycin D (7-AAD; BD Pharmingen, San Jose, CA) for 20 minutes at 4°C. Cells were surface stained as previously described and stained intracellularly with FITC-labeled anti-Ki-67 and APC-labeled anti-BrdU monoclonal antibodies (BD Pharmingen, San Jose, CA) according to manufacturer's instructions.

In vitro migration assays were performed by placing bulk populations of lymphocytes containing predetermined numbers of NK cells (verified by FACs analysis) from the indicated tissues on the top insert of a 5 µm chemotaxis transwell (Fisher Scientific, Waltham, MA) in which the bottom well contained warm media alone or supplemented with 100 ng/mL IL-15c. IL-15c was generated by incubating 100 ng of IL-15 with 500 ng IL-15Rα Fc-chimeric protein at 37° for 20 minutes and 4° for 10 minutes. Plates containing transwells were then incubated at 37°C with CO_2_ exchange, and 90 minutes after plating, cells were harvested from bottom chambers and the percent migration of NK cells was calculated as the ratio of the number of NK cells in the bottom chamber compared to the number of NK cells determined in the input sample.

### Plaque Assays

Plaque assays were preformed as previously described [26]. Briefly, lungs from HK-x31 infected animals were collected and homogenized using a tissue lyser (Qiagen, Hilden, Germany). Monolayers of Madin-Darby kidney cells were incubated with 10-fold serial dilutions of 10% homogenate in dilution media (1×MEM, 1 µg/mL TPCK-treated trypsin) for 1 hour at 37°C. Cells were washed with 1× sterile PBS and overlaid with MEM containing 1.2% Avicel microcrystalline cellulose (FMC BioPolymer, Philadelphia, PA), 0.04 M HEPES, 0.02 mM L-glutamine, 0.15% NaHCO_3_ (w/v), and 1 µg/mL TPCK-treated trypsin. After 72 hours, the overlay was removed, and the cells were washed with 1× sterile PBS, fixed by incubation with cold methanol/acteone (60∶40%), and stained with crystal violet.

### Statistics

Statistical significance was determined by Student's T test using Prism 5 software (GraphPad Software). Significance was determined to be any p-value where p<0.05.

## Results

### NK cells expressing the IL-15 receptor accumulate in the lung airways of influenza-infected animals

We and others have shown that following influenza infection, IL-15 message and protein is increased in the lung airways [19], [27]. Because this IL-15 expression was rapidly induced by influenza infection and reached significant levels as early as day 3 post infection (p.i.) [19], we hypothesized that influenza-induced IL-15 expression may be an important mediator of NK cell responses to influenza infection. We therefore first sought to determine whether NK cells responding to influenza infection were capable of receiving signals from this locally produced IL-15. To this end, lymphocytes were isolated from the lung airways of influenza-infected animals via BAL, and CD3^−^, NK1.1^+^ NK cells were analyzed for the expression of IL-15 receptor components by flow cytometry. The IL-15 receptor is a heterotrimer, consisting of the common gamma chain (CD132), the shared IL-2/IL-15Rβ chain (CD122), and the specific IL-15Rα chain [28]. To date the majority of biological effects of IL-15 on NK cells, however, are mediated through the paired co-expression of CD122 and CD132 [29], [30], [31], while IL-15Rα is only required by accessory cells which present IL-15 to respondent cells, a mechanism referred to as trans-presentation [32]. However, some groups have suggested that IL-15Rα expression alone contains some signaling moieties which may participate in distinct biological functions [27]. Therefore it is important to establish the kinetics of IL-15 receptor component expression and IL-15 signaling potential on NK cells in our model.

**Figure 1:**
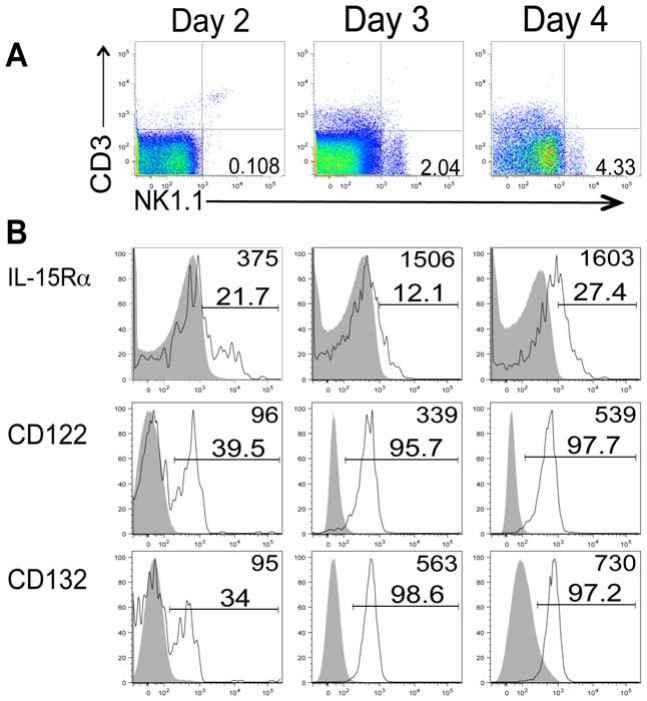
NK cells expressing IL-15 receptor accumulate in the lung airways of influenza-infected animals. Lymphocytes were isolated from the BAL of animals infected with 10^3^ pfu HKx31i.n. at the indicated time points post infection. (A) Representative flow plots of NK 1.1^+^, CD3^−^ cells accumulating in the BAL over time. (B) Expression of IL-15Rα, CD122, and CD132 on NK cells is shown compared to either incubation with the secondary streptavidin alone (for IL-15Rα) or a fluorescence minus one (FMO) control (for CD122 and CD132 expression) (shaded histograms) for each time point p.i. (n = 3 mice). Number in the upper right hand corner represents median fluorescence intensity. Data are representative of two independent experiments.

**Figure 2:**
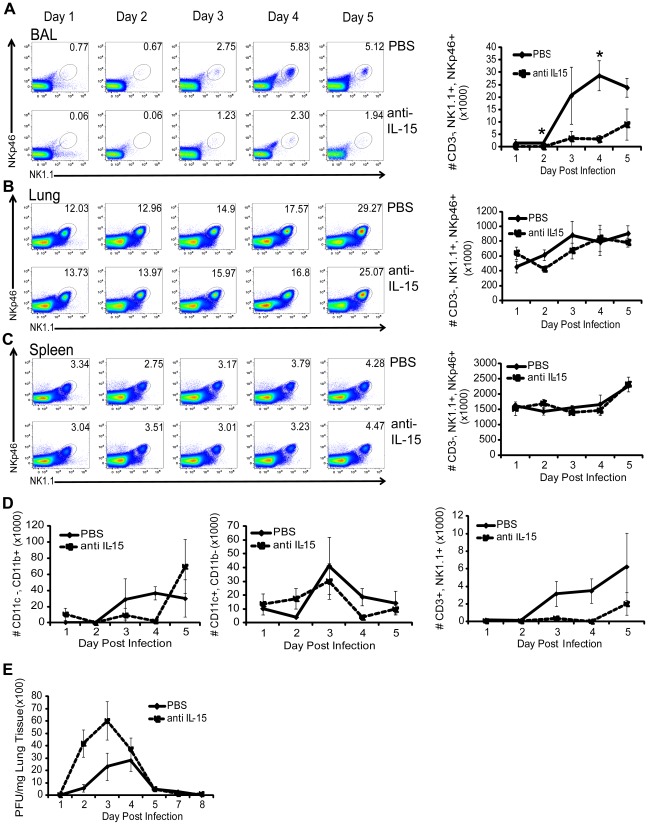
Absence of IL-15 selectively reduces the accumulation of NK cells at the site of infection, which impairs early viral control. Representative flow plots depicting the mean frequency and graphs depicting the mean number ± SEM of CD3^−^, NK1.1^+^ NKp46^+^ NK cells in PBS control (solid lines) or αIL-15-treated (dashed lines) mice recovered from the BAL (A), lung (B), and spleen (C) is plotted over time following i.n. infection with 10^3^ pfu HKx31 (n = 3 mice/group; *p = 0.032 and 0.0145). Data are representative of three independent experiments. (D) Mean number of CD11c^−^ CD11b^+^ cells, CD11c^+^ CD11b^−^ cells, and CD3^+^ NK1.1^+^ cells from BAL are shown ± SEM in PBS control (solid lines) or αIL-15-treated (dashed lines) mice (n = 3 mice/group). (E) At the indicated times p.i. with 10^3^ pfu HKx31, lung viral titers from PBS and αIL-15-treated mice were determined by plaque assay. Mean viral titer is plotted ± SEM (n = 2 mice/group on days 1 and 5 p.i. or 3 mice/group for remaining time points; day 2 p = 0.0503). Data are representative of two independent experiments.

NK cells are known to respond rapidly to influenza infection and continue accumulating in the lung airways through day 5 p.i. ([13] and data not shown). Since we wished to specifically evaluate recent NK cell immigrants responding to the airway inflammation resulting from influenza infection, we restricted our analyses of NK cell kinetics to before and up to day 4 p.i.. NK cells were first detected in the BAL at day 2 post influenza infection (albeit at a low frequency, ∼0.1–0.6% of lymphocytes, Figure 1A and Figure 2A) but were completely absent in control mock-infected animals (data not shown). Despite the low frequency of NK cells in the lung airway at this early time point, one fifth consistently expressed IL-15Rα. Additionally, 30–40% of them expressed CD122 and CD132 (Figure 1B). By day 3 p.i., when NK cells represented a much more discernible population (∼2–3% of lymphocytes, Figure 1A & 2A), greater than 90% of these gated cells expressed CD122 and CD132, and expression levels of these receptors on a per-cell basis increased over time as indicated by a higher median fluorescence intensity by day 4 p.i. (Figure 1B) and consistent with evidence that expression of IL-15R components is induced by activating stimuli [33]. Expression of IL-15Rα however, was variable but consistently much lower that the expression levels of CD122 and CD132. This biased expression of CD122/132 receptor chains over IL-15Rα and enhanced IL-15 levels [19] following influenza infection, indicate that NK cells accumulating at the site of influenza infection are capable of responding to locally produced IL-15 via the trans-presentation pathway.

### IL-15 blockade results in reduced numbers of NK cells at the site of infection and impairs early viral control

Since co-expression of CD122 and CD132 render NK cells responsive to IL-15 signals, we next wished to determine whether virally induced IL-15 and subsequent signaling through these receptors affected the accumulation of these cells in the respiratory tract. Because IL-15^−/−^ mice exhibit a severe developmental defect in both NK and NKT cell lineages and are nearly devoid of these cell populations [20], we chose to use an anti-IL-15 blocking antibody to selectively deplete IL-15 concurrent with infection. Therefore, we monitored the influx of NK cells in animals receiving either PBS or anti-IL-15 mAb administered i.p. daily from day 0 through day 4 post influenza infection. In order to more accurately define bona fide NK cells, particularly in the lung airways harboring few NK cells at very early time points post influenza infection, we included NKp46 reactivity in our staining protocol and henceforth define NK cells as CD3^−^ lymphocytes positive for both NK1.1 and NKp46. While NK cells accumulated in the lung airways of untreated mice as expected, by day 2 p.i. the overall frequency of NK cells in IL-15 blocked animals was reduced by half and remained this low through day 4 p.i. (Figure 2A). Concordantly, total numbers of NK cells in IL-15-blocked mice were partially reduced at day 2 p.i., and substantially diminished by days 3 and 4 p.i. (Figure 2A). In fact, whereas the numbers of NK cells continued to accumulate in the lung airways of control-treated animals through day 4 p.i., numbers of NK cells in the lung airways of treated animals plateaued by day 3 p.i. (Figure 2A). In the lung parenchyma of control-treated animals, NK cells also accumulated over time post infection, similar to those in the lung airways, but the frequencies of NK cells in this site remained unchanged in IL-15-blocked mice. Numbers of NK cells in the lung parenchyma of anti-IL-15 treated animals also remained similar to control animals with only a slight reduction at day 2 p.i. (Figure 2B). Importantly, while the numbers of NK cells were significantly reduced at the site of infection as a result of an IL-15 deficiency, anti-IL-15 treatment had little effect on the frequency or number of NK cells found in anatomical sites distal to the site of infection such as the spleen (Figure 2C). In order to determine whether an absence of IL-15 in the lung airways resulted in the reduced frequencies and numbers of NK cells specifically, numbers of other populations of innate cells in the airways at these early time points post infection were analyzed. CD11c^−^CD11b^+^ cells (granulocytes) or CD11c^+^CD11b^−^ (dendritic cells) in the airways following infection were mostly unaffected by the IL-15 deficiency, with only a small reduction observed at day 4 p.i. (Figure 2D). In contrast, NK1.1^+^CD3^+^ (NKT cells) were markedly reduced in the lung airways of IL-15-blocked mice (Figure 2D), but overall, these cells represented a low proportion of the innate cells responding to influenza infection at these early time points following infection (Figure 1A). Together, these data indicated that short term blockade of IL-15 did not result in global defects in NK cell homeostasis or survival in peripheral tissues and the effects of IL-15 were largely specific to NK cells as blocking IL-15 selectively resulted in a significant loss of NK cells recovered from the site of infection.

To test whether this local reduction in NK cells impairs the control of viral replication, we performed plaque assays on control- and anti-IL-15 treated mice to quantify viral load in the lungs of animals with intact or diminished IL-15 and NK cell responses. Viral load was quantified on days 1–5 and 7–8 p.i. to specifically look at control during the time frame of NK cell entry and accumulation in the lung airways and the kinetics of subsequent viral clearance. In IL-15 blocked animals, differences in viral load were apparent as early as d2 p.i. where viral titers were about 3× higher through day 3 p.i. (Figure 2E); however, these animals seemed to regain control of viral replication by day 4 p.i., which perhaps corresponds with the early entry of cells of the adaptive immune response as anti-influenza specific CD8 T cells are first detectable in the lung airways by d6 post infection by flow cytometry ([34] and data not shown). Thus, while viral elimination is not ultimately dependent on IL-15, early control of the virus is impaired in the absence of IL-15, which correlates with the arrival of a significant number of NK cells in the lung airways. We thus hypothesized that IL-15 was important for the migration of NK cells in the lung airways following influenza infection.

### Administration of exogenous IL-15 enhances the number of NK cells in the lung airways

We observed significant reductions in the numbers of NK cells in the lung airways of influenza-infected animals in which IL-15 was blocked at time points associated with their arrival at the site of infection, and failure of these cell populations to accumulate had implications in early viral control (Figure 2). In order to determine whether IL-15 might be an important signal for NK cells in the migration to and/or the proliferation within the site of infection, we chose to provide exogenous IL-15 in an attempt to enhance any IL-15-dependent NK cell migration and/or in situ proliferation within the lung airways of influenza-infected animals. To this end, either PBS or recombinant IL-15/IL-15Rα fusion protein complexes (IL-15c) were administered intranasally to mice three days following influenza infection. To ensure that any biological effects of the IL-15c could be attributed to activity of the cytokine (which is merely stabilized by complexing to IL-15Rα), a control group of mice received the IL-15Rα only. Concurrent with treatment, mice received an i.p. pulse of the thymidine analog BrdU to identify proliferating cells. Twelve hours post treatment, the frequency and total number of NK cells in the BAL was quantified as well as the percentage of these cells incorporating BrdU. Isolated cells were simultaneously stained with 7-AAD as an indicator of cell viability, as only cells with disrupted membranes stain positive for this fluorescent dye. Importantly, neither the IL-15Rα alone nor the IL-15c affected cell viability, as cells isolated from animals receiving these treatments had similar percentages of 7-AAD^+^ NK cells as those from PBS-treated mice (data not shown).

**Figure 3:**
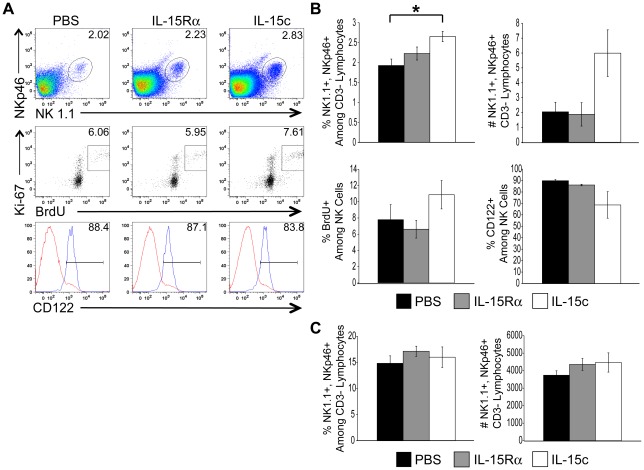
Administration of IL-15c i.n. increases the number of NK cells recovered from the lung airways. On day 3 p.i. with 10^3^ pfu HKx31, mice were administered either PBS vehicle control (black bars), IL-15Rα alone (shaded bars) or IL-15c (open bars) intranasally. Twelve hours post treatment, CD3^−^ lymphocytes were analyzed for NK1.1 and NKp46 expression. These NK cells were quantified and analyzed for CD122 expression, BrdU incorporation, and Ki-67 staining. Representative flow plots are depicted in panel (A), and graphical representations of mean frequencies and numbers of NK cells ± SEM, as well as percentage of these cells positive for CD122 and BrdU are depicted in panel (B) (n = 3 mice/group; * = p<0.025). (C) Mean frequencies and numbers of NK cells in the spleen are plotted ± SEM (n = 3 mice/group). Data are representative of two independent experiments.

Upon introduction of IL-15c to the lung airways, the overall frequency of NK cells isolated from the BAL was significantly increased (Figure 3A and B), and the total number of NK cells isolated from this site was nearly three times that of PBS-treated control animals (Figure 3B). Interestingly, the percentage of NK cells expressing CD122, the IL-2/15 Rβ chain, was reduced in IL-15c-treated animals (Figure 3B), perhaps indicative of increased signaling through and subsequent internalization of this receptor complex by IL-15 responsive cells. Unlike T cells, which require large clonal bursts of proliferation to achieve effector status, the effector function of NK cells is more related to activation and mobilization to the site of inflammation [35]. Nevertheless, we wished to test whether the large increases in NK cell number in the lung airways following IL-15c administration could be attributed to IL-15-induced proliferation of NK cells at the site. To assess the potential role of proliferation in this observed increase in NK cell frequency and number in the BAL of treated mice, these NK cells were analyzed for BrdU incorporation. Concomitantly, cells isolated from the BAL were assessed for expression of the cell-cycle-specific protein Ki-67. Since BrdU is incorporated into the DNA of only cells in S phase whereas Ki-67 is expressed by cells in any stage of the cell cycle, it was unsurprising that BrdU^+^ cells were only a fraction of Ki-67^+^ cells (Figure 3A). Therefore, we considered only cells positive for both markers as cells undergoing proliferation at the time of treatment. Although the percentage of BrdU-incorporating cells was modestly increased in IL-15c-treated animals, the overall frequency of proliferating cells was low in untreated (<10%) and treated (<12%) animals (Figure 3B). These data suggest that IL-15c may trigger the proliferation of NK cells, but proliferation of this cell population in the lung airways is, in general, low and not likely to be solely responsible for the total number of cells extracted from the lung airways. IL-15Rα alone had no significant impact on the accumulation or proliferation of this cell population (Figure 3A and B). Finally, differences in cell frequencies, numbers, CD122 expression, and BrdU incorporation were specific to the lung airways (the sight of treatment), as NK cells isolated from spleens were similar in control and IL-15c-treated animals (Figure 3C and data not shown). Together, these data demonstrate that exogenous IL-15 results in increased numbers of NK cells in the lung airways. Since this increase appeared to be independent of IL-15-mediated effects on cell survival, and proliferation was low, we hypothesized that IL-15 may be responsible for a substantial amount of migration of NK cells into the lung airways following influenza infection similar to its effects on CD8 T cells [19].

### NK cells migrate to IL-15c in vitro

**Figure 4:**
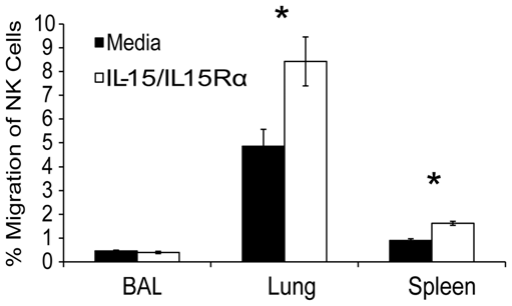
IL-15 is chemotactic for NK cells in vitro. Three days following infection with 10^3^ pfu HKx31 i.n., 1×10^6^ bulk lymphocytes from the pooled BAL, lung, and spleen of 8 mice were placed in the top chamber of a transwell with the bottom chamber containing 500 µL either media alone (black bars) or supplemented with 100 ng IL-15c (open bars). Mean percent migration of CD3^−^, NK1.1^+^ NK cells is depicted ± SEM (n = 3 replicates/group; *p = 0.046 and 0.003). Data are representative of three independent experiments. Significant differences in migration to media alone or media containing IL-15c are indicated by stars (*p<0.05).

Intranasal administration of IL-15c resulted in increased numbers of NK cells in the lung airways that appeared to be due to increased migration into that site. Previous studies have indicated that IL-15 is indeed chemotactic for NK cells. In vitro checkerboard assays revealed that freshly isolated NK cells migrated to IL-15 gradients, and IL-15 stimulation increased LFA-1-dependent binding of NK cells to cultured endothelial cells [25]. IL-15 has also been shown to play a central role in the recruitment of CD16^−^ human NK cells into the endometrium following ovulation [36]. To test the direct chemotactic potential of IL-15c for NK cells in our own system, we employed an in vitro chemotaxis transwell assay. Bulk lymphocytes (or purified splenic NK cells, data not shown) from the BAL, lung, and spleen of mice collected three days after infection with influenza were placed in the top chamber of a transwell filter support with either media alone or media supplemented with IL-15c in the bottom chamber. After 90 minutes of incubation, IL-15c significantly enriched NK cells isolated from the lung and spleen (Figure 4A). Consistent with our findings with CD8 T cells [19], NK cells from the lung airways did not migrate to IL-15c, perhaps because this site represents the terminal destination for these cell populations. Unlike CD8 T cells, which lose expression of CD122 upon residence in the lung airways following influenza infection [19] [37], nearly 100% of the NK cells residing in the BAL of influenza-infected mice express CD122 (Figure 1A and B). Nonetheless, these data indicate that NK cells in the lung parenchyma or the general circulation (as represented by the spleen) migrate to IL-15 in vitro.

### Intranasal administration of IL-15c during the innate phase enhances early viral control

**Figure 5:**
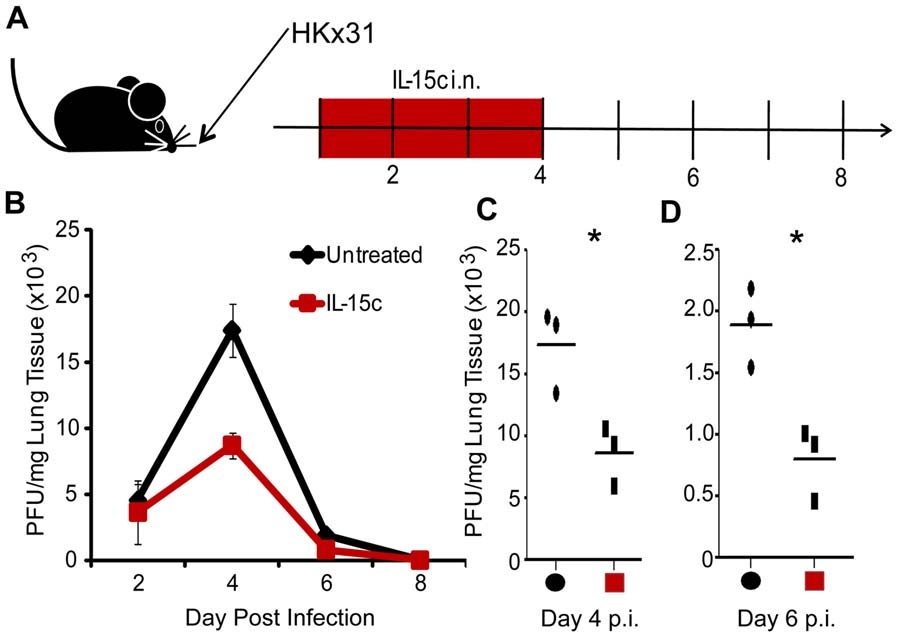
Intranasal administration of IL-15c during the innate phase of the immune response against influenza improves early control of viral control. On days 1–4 post infection with 10^3^ pfu HKx31 i.n., animals received either PBS vehicle control (Black circles) or IL-15c (red squares) i.n. (A). Whole lungs were collected and analyzed via plaque assay for viral titer on days 2, 4, 6, and 8 p.i. (B). Significant differences between control and IL-15c-treated mice were observed on days 4 (C) and 6 (D) p.i. (*p = 0.022 and 0.013).

A temporal cessation in IL-15 bioavailability reduced the numbers of NK cells (Figure 2A) in the respiratory tract resulting in increased viral titers (Figure 2E), presumably due to impaired NK cell responses. Conversely, exogenous IL-15c promoted the migration of NK cells (Figure 4) and resulted in increased numbers of NK cells in the lung airways (Figure 3B). We therefore hypothesized that intranasal administration of IL-15c early after infection could be used to enhance the early innate immune response to influenza and augment viral control. To this end, influenza-infected animals received either PBS or IL-15c intranasally on days 1–4 p.i., a time frame corresponding to the migration of NK cells into the lung airways and limiting any confounding effects IL-15c might have on adaptive immune cells entering the lung airways at later time points. Every other day, from day 2–8 p.i., whole lungs were collected and viral titers were quantified via plaque assay (Figure 5A). Early (d2) p.i., we observed no difference in viral load between PBS and IL-15c-treated mice, but as viral replication reached more significant levels at day 4 p.i., animals receiving the IL-15c had 2.4× less viral load than animals receiving only PBS (Figure 5B and C). Although viral titers dropped 10 fold in both groups of animals by day 6 p.i., as expected, surprisingly, there remained a greater than 2 fold significant difference in viral load (Figure 5B and D). Although both groups of animals cleared the influenza virus completely by day 8 p.i. (Figure 5B), our data suggest administration of IL-15c on days 1–4 p.i. can enhance early control of influenza virus by cells of the innate immune system.

### NK cell responses to influenza are requisite for the optimal accumulation of antigen-specific CD8 T cells at the site of infection

**Figure 6:**
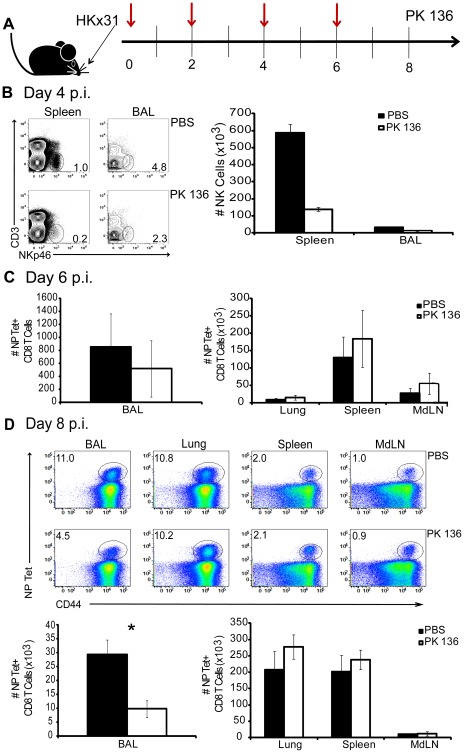
NK1.1+ NK cells are partially required for the subsequent accumulation of influenza-specific CD8 T cell accumulation at the site of infection. Beginning on the day of infection, mice received i.v. injections of either PBS vehicle control or αNK1.1 (PK136) every other day until day 6 p.i. (red arrows), and indicated tissues were collected on days 4, 6, and 8 p.i. (A). Mean number NK cells cells on day 4 p.i. in untreated (black bars) and PK136-treated (open bars) are shown ± SEM (n = 3 mice/group) (B). Mean number of NP-Tet^+^ CD8 T cells in the BAL, lung, and spleen were quantified and depicted ± SEM on day 6 (C) and day 8 (D) p.i. (n = 3 mice/group). Stars indicate statistical significance (*p = 0.009). Data are representative of three independent experiments.

Because we have previously reported that IL-15 is important for the migration of influenza-specific CD8 T cells into the lung airways [19], it was possible that those observed effects were secondary to the recruitment of NK cells to the lung. To test whether the accumulation of NK cells in the lung was required for the subsequent immigration of influenza-specific CD8 T cells to the respiratory tract, influenza-infected animals were assayed for the accumulation of anti-influenza specific CD8 T cells in NK deficient animals, generated by administration of the αNK1.1 depleting mAb PK136 every other day (Figure 6A). Flow cytometric analyses of NKp46 expression of day 4 p.i. lymphocytes isolated from the lung, BAL, spleen, and mediastinal lymph nodes (MdLN) of αNK1.1-treated animals revealed robust depletion of NK cells as the number of CD3^−^NKp46^+^ cells were reduced to less than one third of those observed in PBS-treated control animals (Figure 6B and data not shown). On days 6 and 8 p.i., the numbers of influenza-specific CD8 T cells in these same tissues were quantified through the identification of cells staining positive for a tetrameric reagent loaded with the immunodominant peptide derived from the influenza nucleoprotein (NP). Tetramer positive CD8 T cells could first be detected in the lung airways at day 6 p.i., and although numbers were low, they were somewhat reduced in the BAL of PK136-treated mice (Figure 6C). No reduction in influenza-specific CD8 T cell numbers could be observed in any other tissue. In fact, CD8 T cells seemed to accumulate in other tissues of animals depleted of NK1.1-expressing cells. By day 8 p.i., the frequency of influenza-specific CD8 T cells in the BAL of PK136-injected animals was less than half than that of control animals, and the total numbers were reduced nearly threefold (Figure 6D). Again, this effect was specific to the lung airways, since frequencies and numbers of NP- tetramer^+^ CD8 T cells were unchanged in other tissues examined (Figure 6D). Therefore, the largely tissue-specific nature of the dependence of influenza-specific CD8 T cells on the presence of NK1.1-expressing cells in the lung airways partially indicates that lung-resident NK and possibly NKT cells are requisite for the subsequent migration of influenza-specific CD8 T cells into this site. These data suggest that IL-15-mediated migration of CD8 T cells to the lung airways may be, at least partially, an indirect effect of NK1.1^+^ cells moving into the lung airway space in response to IL-15 and that subsequent tissue remodeling or production of an intermediate factor is responsible for the subsequent recruitment of the CD8 T cells. To our knowledge, these data for the first time describe a role for IL-15 in linking the innate and adaptive responses to influenza infection necessitating the further inquiry of IL-15 as a potential vaccine adjuvant.

## Discussion

Here, we have demonstrated that NK cells, which accumulate in the lung airways early after influenza infection, are dependent on IL-15 for this accumulation and subsequent ability to control viral load. NK cells in the lung airways express high levels of the common gamma chain (CD132) and CD122, receptors responsible for imparting IL-15 responsiveness. Since IL-15 is known to be produced in the lung airways following influenza infection, we investigated the role of IL-15 in NK cell responses to influenza. In the absence of IL-15, NK cell frequencies and numbers were significantly reduced in the BAL, resulting in impaired early control of influenza virus. Although numbers of CD3^+^ NK1.1^+^ cells were also substantially reduced following anti IL-15 treatment, it is unclear whether this cell population plays a relevant role in controlling primary infections with influenza. While NK cells are thought to be very important participants in the control of influenza replication, evidence for NKT cells playing a similar role is more controversial [38]. Models of NKT cell activation using αGal-Cer revealed enhanced innate responses to influenza and improved disease outcome [39], but challenge of CD1d^−/−^ mice with influenza led to increased survival, implying an immuno-regulatory role for NKT cells in this model [40]. Whether or not these cells are critically involved in viral clearance, in our model, they represent a very small percentage (<1%) of the lymphocytes in the lung airways in the first five days following infection. Therefore, we believe that an abrogation of virally-induced IL-15 in the lung airways most dramatically affects CD3^−^, NK1.1, NKp46 double positive NK cells responding rapidly to infection. By the same principle, exogenous IL-15 could be used therapeutically to increase NK cell populations in the BAL—primarily as a result of IL-15-induced migration—to enhance viral control. Therefore, these combined data indicate that influenza-induced IL-15 is an important signal for the migration of NK cells to the lung airways where they help limit viral replication.

IL-15 is produced by a variety of cell types within and outside of the immune system, including dendritic cells, monocytes and macrophages, stromal cells, endothelial cells, and epithelial cells [41], and pathogenic stimuli are known to induce this expression above constitutive levels [17], [42], [43], [44]. Although dendritic cells are known to be an important source of IL-15 at day 6 post influenza infection [27], it is unclear whether DCs or other cell types produce IL-15 to facilitate specific immune responses to influenza. For example, lung epithelial cells constitutively express IL-15 and IL-15Rα [45], and neutrophils and macrophages can be a major source of IL-15 that is produced during a variety of lung inflammatory diseases including sarcoidosis, tuberculosis, bronchitis, and asthma [46]. Following influenza infection, the IL-15-producing cell type(s) is/are still unknown, but influenza-induced expression of IL-15 is clearly an important regulator of the NK and CD8 T cell responses to this virus [19].

Not only might there be different cellular sources of IL-15 at different times after infection, but IL-15 signaling in NK cells could also be regulated by the context in which IL-15 is presented. At least one isoform of IL-15 is known to enter the cell secretory pathway and could therefore be released as a soluble molecule [47], [48]. These data lend the idea that soluble IL-15 could bind heterotrimeric receptors on NK cells. However, IL-15 is also known to be transpresented to NK cells, and indeed, this mode of signaling appears to be most important for IL-15-mediated NK cell survival [49]. While IL-15Rα is dispensable for NK cell survival, IL-15Rα is thought to be an important component in signaling the migration of CD16^−^ human NK cells into the endometrium, since this particular NK cell population expresses higher levels of this receptor component than CD16^+^ NK cells that do not migrate to IL-15 [36]. In our studies, IL-15Rα was only expressed on a relatively low proportion of NK cells in the lung airways of influenza-infected animals (Figure 1). In contrast, CD122 signaling appears to be important for NK cell migration to IL-15 as this receptor chain is down regulated on NK cells exposed to IL-15c in vivo (Figure 3). Thus, we consider it likely that signaling through IL-15Rα is not essential to IL-15-mediated of migration of NK cells to the lung airways. Therefore, future work is needed to identify the IL-15-producing cell population(s) and the mechanism of migration to this cytokine in order to target this response for eventual applications in influenza vaccines and treatments.

A significant finding of our work is that NK cells immigrating into the lung airways are partially required for substantial trafficking of influenza-specific effector CD8 T cells to this location. We found that NK cells are necessary for the optimal accumulation of antigen-specific CD8 T cells, important effectors of eventual viral clearance [4], [5], at the site of infection, since depletion of NK1.1-expressing cells resulted in a significant decrease in the number of influenza-specific CD8 T cells in the BAL. A subpopulation of CD8 T cells has been shown to express NK1.1 upon activation [50], and while these cells were detected in the lung parenchyma of influenza-infected C57Bl/6 mice, they comprised less than 1% of the NP-tetramer+ CD8 T cells at this site alone and were never detected in the lung airways. This and the fact that the reduction of activated, influenza-specific CD8 T cells was observed only in the lung airways, and only at later time points post infection lead us to believe that direct depletion of NK1.1-expressing CD8 T cells cannot account for this reduction in treated mice. These data strongly suggest that NK1.1^+^ cells in the lung airways are requisite for the subsequent accumulation of influenza-specific CD8 T cells in the BAL and implicate IL-15-mediated NK cell migration into the lung airways as a link between the innate and adaptive responses to influenza infection. We currently favor a model in which NK cells migrate directly to influenza-induced IL-15 in the lung airways, and, CD8 T cell trafficking, in turn, occurs to both IL-15 directly and to chemotactic factors produced by NK cells already present at the site. A positive feedback loop in which NK cells responding to IL-15 induce further expression of IL-15 by dendritic cells for the stimulation of CD8 T cells is an additional possibility [51]. Moreover, IL-15 has also been shown to modulate chemokine and chemokine receptor expression by NK cells and T cells [52], [53], [54]. As IL-15 deficiencies also cause reductions in CD8 T cell accumulation in the BAL [19], chemotactic potential of IL-15 for NK cells presented here provides a possible link between IL-15-mediated effects of both the innate and adaptive immune responses to influenza infection. Future work will attempt to tease out the roles of direct and indirect migration of both NK cells and CD8 T cells to IL-15 following influenza infection.

Regardless of mechanism, IL-15-induced trafficking of lymphocytes is an important part of our overall understanding of influenza immunobiology. The studies presented here emphasize the importance of IL-15 in mediating the innate response to influenza via the trafficking of NK cells, as viral titers were not as efficiently controlled early after infection in IL-15-blocked animals. These and previous studies also suggest that exogenous IL-15c could be used to modulate the immune response at both the innate and adaptive phases. Overall, we believe that IL-15 may be in important point of eventual immune intervention for treatment of primary infection and for adjuvanting vaccines.
